# Characteristics of NAFLD Based on Hypopituitarism

**DOI:** 10.1155/2020/8814435

**Published:** 2020-10-10

**Authors:** Kazuhisa Kodama, Atsuhiro Ichihara, Yasufumi Seki, Yuichi Ikarashi, Takaomi Sagawa, Tomomi Kogiso, Maiko Taniai, Katsutoshi Tokushige

**Affiliations:** ^1^Department of Gastroenterology and Medicine, Tokyo Women's Medical University, Tokyo, Japan; ^2^Department of Medicine II, Endocrinology and Hypertension, Tokyo Women's Medical University, Tokyo, Japan

## Abstract

**Background:**

Hypopituitarism and hypothalamic disorders, which induce central obesity and appetite disorder, are associated with nonalcoholic fatty liver disease (NAFLD). We retrospectively analyzed the clinical features of NAFLD patients with hypopituitarism. *Patients.* We examined the cases of 15 NAFLD patients with hypopituitarism (mean age, 39.4 years; males/females, 11/4). The causes of hypopituitarism were surgical in eight cases (six with craniopharyngioma and two with prolactinoma) and nonsurgical in seven cases, including unexplained hypopituitarism in five cases, Sheehan syndrome in one case, and one case that occurred after the radiation therapy. Serum adiponectin, soluble tumor necrosis factor receptor-2 (TNFR-2), and leptin levels were measured.

**Results:**

We compared the cases of the eight patients who underwent cranial surgery due to craniopharyngioma or prolactinoma and seven nonsurgical cases. The body mass index (surgery group, 30.2 ± 4.1; nonsurgery group, 29.2 ± 14.2) and the rate of diabetes (75% in surgery group, 14.3% in nonsurgery group) tended to be higher in the surgery group, and the hepatic fibrosis grade (surgery group, 3.75 ± 0.38; nonsurgery group, 1.64 ± 1.07) was significantly higher in the surgery group. The levels of adipocytokines, serum adiponectin, and serum soluble TNFR-2 showed no correlation with hepatic fibrosis, whereas the serum leptin levels were significantly correlated with liver fibrosis (*R* = 0.696).

**Conclusion:**

The hepatic fibrosis grade rapidly progressed in the cranial surgery cases of NAFLD patients with hypopituitarism, possibly in association with BMI, diabetes mellitus, and leptin. In such cranial surgery patients, strong interventions should be considered from the early stage, including diet education, hormone replacement, and more.

## 1. Introduction

Nonalcoholic fatty liver disease (NAFLD) has become the most common type of liver disease in developed countries worldwide. NAFLD covers a wide spectrum from nonalcoholic fatty liver (NAFL) to nonalcoholic steatohepatitis (NASH), which can progress to cirrhosis and hepatocellular carcinoma [1–3]. NAFLD is closely associated with obesity, lifestyle, and lifestyle-related diseases [1–5].

Hypopituitarism, resulting in central obesity, is frequently known to lead to a condition similar to metabolic syndromes such as hyperlipidemia, impaired glucose tolerance, and fatty liver [6]. It has been reported that in patients with NAFLD and lifestyle-related diseases based on growth hormone (GH) deficiency associated with hypopituitarism, these metabolic conditions and liver function were improved by the administration of GH [7]. In addition, thyroid dysfunction is observed in approximately 25% of patients with NAFLD [8]. These findings suggested that endocrine hormonal abnormalities are closely related to the development of NAFLD.

The hypothalamus, where leptin receptors are present, is closely related to appetite control and to adjustments of the sympathetic nervous system, and thus, the relationship between hypothalamic disorders and NAFLD is also attracting attention [9]. These findings suggested that hypopituitarism and hypothalamic disorders are closely related to the development of NAFLD.

Based on the above reports, we examined the clinical features of NAFLD patients with hypopituitarism and/or hypothalamus disorder.

## 2. Patients and Methods

### 2.1. Patients

We collected the data of the 15 patients with NAFLD with hypopituitarism who were diagnosed based on findings obtained by a liver biopsy, blood test, endocrine test, CT scan, or other modes between January 2000 and December 2019 at our institute for a retrospective analysis. This study conformed to the ethical guidelines of the Declaration of Helsinki (2000 version) and was approved by our institute's ethics committee. Regarding informed consent, if we were able to contact the patients, we obtained their informed consent directly. For the other cases, we posted the study plan on our institute's home page. If any patients or bereaved family members refused their consent for this study project, we deleted the patient's data.

### 2.2. Diagnosis and Management of NAFLD with Hypopituitary Dysfunction

The diagnosis of NAFLD was based on the following criteria: (1) the detection of hepatic steatosis (or steatohepatitis) by liver biopsy (steatosis was diagnosed in >5% of liver biopsies [10]); (2) ethanol intake <20 g/day in women or <30 g/day in men (confirmed by the attending physician and/or family members in close contact with the patient); and (3) appropriate exclusion of other liver diseases. Other liver diseases, such as alcoholic liver disease, viral hepatitis, autoimmune hepatitis, drug-induced liver disease, primary biliary cirrhosis, primary sclerosing cholangitis, biliary obstruction, and metabolic liver diseases such as Wilson's disease and hemochromatosis were excluded on the basis of patient interviews regarding ethanol intake, blood chemistry tests, virus markers, auto-antibody, ultrasound, and CT scans [2, 10].

The diagnoses of hypopituitarism and hormone replacement therapy were performed by the department of medicine II, endocrinology, and hypertension at our hospital. Briefly, several tests were performed for the diagnosis of hypopituitarism: (i) blood findings, thyroid hormone levels, and adrenal and sex hormones were measured. (ii) Several hormone stimulation tests matched to the patient's hormone levels were conducted (TRH stimulating test and ACTH stimulating test). (iii) The results of brain imaging magnetic resonance imaging (MRI) or high-resolution CT and pituitary tumor or other pituitary gland problems were examined. (iv) Vision tests: we investigated whether each patient's sight or visual field was impaired. In some patients' cases, to compensate for hormone deficiencies, hormones such as GH, glucocorticoid, thyroid hormone, antidiuretic hormone, and/or sex hormones were administered, matched to the individual patient's hormone level.

The date of the liver biopsy was taken as the time of the patient's diagnosis of NAFLD. Each patient's body mass index (BMI), liver enzymes, and lipid and glucose profiles were obtained at the time of liver biopsy thereafter. The diagnosis of type II diabetes mellitus was based on the World Health Organization criteria. Liver histology was reviewed by liver pathologists and was evaluated according to the modified classification published by Brunt [11, 12]. Fibrosis was scored using a five-grade scale: F0, normal connective tissue; F1, perivenular or pericellular fibrosis in zone 3; F2, perivenular or pericellular fibrosis with focal or extensive portal/periportal fibrosis; F3, bridging or septal fibrosis; and F4, cirrhosis. The inflammatory grade and steatosis grade were also evaluated.

After the liver biopsy, enzyme-linked immunosorbent assays were used to measure the patient's serum levels of adiponectin (Otsuka Pharmaceutical, Tokyo), soluble tumor necrosis factor receptor-2 (TNFR-2, Biosource Europe, Fleurus, Belgium), and leptin (AssayPro, St. Charles, MO, USA).

### 2.3. Statistical Analyses

The patients' data were analyzed with SPSS version 13.0J software (SPSS, Tokyo). The results are shown as median values or percentages. The Mann–Whitney test or chi-square test was performed to detect significant differences between the data of the cranial surgery NAFLD and nonsurgery NAFLD groups. The correlations between the hepatic fibrosis grade and the serum soluble TNFR-2 level, serum adiponectin level, and serum leptin level were examined by Spearman's correlation test. The correlation index (*R*) was calculated. In all analyses, probability (*p*) values <0.05 were considered to indicate the significance.

## 3. Results

As shown in Table 1, the mean age of the total series of 15 patients at the time point of their liver biopsy was 39.4 years old and the male/female ratio was 11/4. The patient's mean age at the diagnosis of hypopituitary dysfunction was 18.5 years old, and the duration from the diagnosis of hypopituitarism to the liver biopsy was 20.7 years.

The causes of hypopituitarism are also shown in Table 1. There were eight surgery cases (six patients with craniopharyngioma and two with prolactinoma) and seven nonsurgery cases, including five patients with unexplained pituitary dysfunction, one patient with Sheehan syndrome, and one patient with hypopituitarism after the radiation therapy. Thyroid hormone supplementation therapy was administered to all 15 patients, glucocorticoids were given to 12 patients (80%), and five patients received GH (33%). Regarding the liver pathological findings, all 15 patients showed steatohepatitis. The fibrosis grade was 2.80 ± 1.33, the inflammation grade was 1.90 ± 0.63, and the steatosis grade was 2.10 ± 0.89.

Based on our finding that all eight surgery patients (with craniopharyngioma or prolactinoma) showed a fibrosis grade of F3 or F4, we compared the eight surgery patients and seven nonsurgery patients (Tables 2–4). Although there was no significant between-group difference in age or gender, the surgery group tended to have higher BMI values (surgery group 30.2 ± 4.1 vs. nonsurgery group 29.2 ± 14.2, *p*=0.072) and a higher rate of diabetes (75% in the surgery group vs. 28.6% in the nonsurgery group, *p*=0.072). The platelet count and serum cholesterol level were significantly lower in the surgery group. Regarding liver pathological findings, the fibrosis grade was significantly more severe in the surgery group at 3.75 ± 0.38 versus the nonsurgery group's grade at 1.64 ± 1.07 (*p*=0.01). The activity grade and steatosis grade were not significantly different between the surgery and nonsurgery groups. We confirmed the abovementioned significant differences using a multivariate unconditional logistic regression model. The between-group differences in the hepatic fibrosis grade (*p*=0.01), serum albumin level (*p*=0.048), platelet count (*p*=0.04), and total cholesterol level (*p*=0.012) were confirmed to be significant.

**Figure 1:**
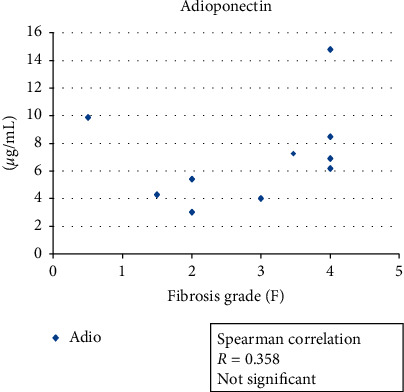
The association between the serum adiponectin level and hepatic fibrosis grade in 10 NAFLD patients with hypopituitarism. Spearman's correlation was obtained. There was no significant association (*R* = 0.358).

**Figure 2:**
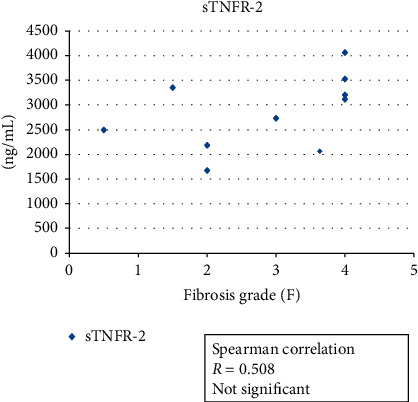
The association between the patients' serum soluble TNFR-2 level and hepatic fibrosis grade (*n* = 10). Spearman's correlation was obtained. There was no significant association (*R* = 0.508).

**Figure 3:**
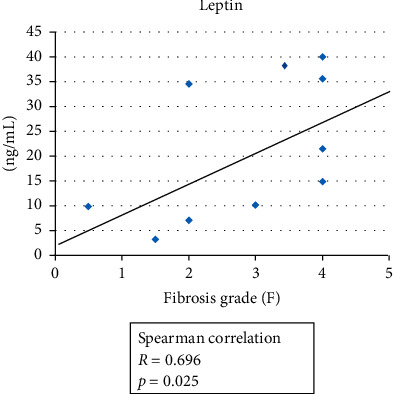
The association between the patients' serum adiponectin level and hepatic fibrosis grade (*n* = 10). Spearman's correlation was obtained. A significant association was observed (*p*=0.025, *R* = 696).

For our investigation of the associations of serum adiponectin values and serum leptin values with serum soluble TNFR-2 levels, we examined 10 of the 15 patients' values and determined their correlation with liver fibrosis. The results revealed no correlation of the serum adiponectin level or the serum soluble TNFR-2 levels with the hepatic fibrosis grade. In contrast, these patients' serum leptin levels showed a significant correlation with their liver fibrosis grades (*R* = 0.696, *p*=0.025) (Figures 1–3).

## 4. Discussion

The importance of NAFLD due to hypopituitarism has been described, as such cases of NAFLD can rapidly progress to cirrhosis even at a young age [13–15]. We have treated a number of these cases. In cases of GH deficiency associated with hypopituitarism, the prevalence of liver fat deposition in the abdominal echo is generally approximately 60%; the frequency of fatty liver is high compared to that in general populations [6]. Growth hormone was administered to the patients with lifestyle-related disease, and it enhanced their metabolism and energy consumption [6, 7]. The improvement of liver function after GH administration is considered to be due to improvements in the patient's metabolic and energy consumption state [7, 16]. Growth hormone is also expected to be useful as a treatment strategy aimed at the metabolic and energy improvement of general NAFLD patients in the future. A multicenter trial was performed to investigate the efficacy and safety of tesamorelin (a synthetic form of GH-releasing hormone) in NAFLD patients with HIV, and it was reported that tesamorelin treatment resulted in a greater reduction of the hepatic fat fraction [17].

In our present liver biopsy cases—specifically in the patients who underwent surgery due to craniopharyngioma or prolactinoma—it became clear that liver fibrosis had progressed. We speculate that this progression of liver fibrosis impaired not only the pituitary gland but also the hypothalamus, due to the presence of a large tumor or surgical resection. As the hypothalamus is involved in the appetite center and in the adjustments of the sympathetic nervous system, it is known that eating disorders and decreased sympathetic nerve activity are induced in addition to GH deficiency. The BMI values of our present surgery cases tended to be higher than those of the nonsurgery group, suggesting that the surgery patients' cases might have been associated with eating disorders due to a hypothalamus disorder.

In addition, leptin is produced by increased fat cells due to obesity, and it usually works in the hypothalamus to control the feeding center. However, in the present surgical cases, leptin would not have been functioning in the hypothalamus or feeding center. Ikejima et al. reported that leptin enhanced liver fibrosis [18, 19], and we thus speculate that in the present study's patients who underwent surgery, leptin caused the liver fibrosis to rapidly progress without appetite control. The significant correlation that was revealed between the serum leptin levels and liver fibrosis supports this speculation. Therefore, in these cranial surgical cases involving the pituitary gland and hypothalamus, the appearance of NAFLD had to be closely monitored. We recommend extensive diet therapy and hormone replacement therapy from an early stage for such patients.

We also measured other adipokines (i.e., adiponectin and soluble TNFR-2 instead of TNF*α*). Several papers have demonstrated that both of these adipokines have an important role in progression and liver fibrosis in NASH/NAFLD [20–22]. In addition, Bach et al. reported that pituitary function is decisive for the catabolic response to TNF*α* [23]. Ames dwarf mice (which are GH-deficient) showed a beneficial adipocytokine profile, characterized by increased adiponectin and decreased proinflammatory cytokine (TNF*α* and interleukin-6) levels [24]. We therefore measured the present patients' levels of these two adipokines; however, we could not observe a significant correlation between the liver histological findings in NAFLD based on hypopituitarism.

We reported the case of the present patient #15 with idiopathic hypopituitarism and hepatopulmonary syndrome due to NASH [25]. After the administration of GH, the patient's NASH and hepatopulmonary syndrome were improved. Neither an appetite disorder nor obesity was observed in this patient, suggesting that his hypothalamus was intact and that the GH was thus effective. The treatment of patients with hypothalamic disorders might require another method in addition to the GH hormone therapy. In fact, it has been difficult to enforce dietary restrictions with normal dietary guidance for these patients. The elucidation of a new central appetite system other than the leptin-hypothalamic system and the development of an appetite suppression mechanism are expected.

Bariatric surgery is now also considered a treatment option for patients with NAFLD who find it difficult to control their appetite and body weight [26]. In our present patient series, bariatric surgery might have been an effective therapy method for appetite control; however, the safety and efficacy of bariatric surgery for patients with NASH cirrhosis have not been established. Even in NAFLD cases associated with metabolic syndrome, the involvement of glucocorticoids, GH, thyroid hormones, and a loss of appetite control, i.e., so-called leptin resistance, should be considered.

This study has several limitations. It was a single-center retrospective analysis of a small number of patients (*n* = 15). Multicenter and prospective studies are desired for the further elucidation of the findings obtained herein. In conclusion, hepatic fibrosis progresses rapidly in NAFLD patients with not only hypopituitarism but also hypothalamus dysfunction, and this might be associated with BMI, diabetes mellitus, and leptin.

## Figures and Tables

**Table 1:** Clinical features of NAFLD with hypopituitarism.

Case	Gender	Age at diagnosis of hypopituitarism (yrs)	Age at liver biopsy diagnosis (yrs)	Cause of hypopituitarism	Surgery	Hormone replacement therapy^*∗*^
1	M	5	16	Craniopharyngioma	〇	G, S, T, and A (+radiation)
2	M	6	18	Craniopharyngioma	〇	S, T, and A
3	M	6	44	Craniopharyngioma	〇	S, T (+gamma knife)
4	M	9	24	Idiopathic hypopituitarism	X	T and A
5	M	10	18	Transection of the pituitary stalk	X	G, S, and T
6	M	12	43	Germ cell tumor	X	S and T
7	M	12	50	Idiopathic hypopituitarism	X	G and T
8	F	13	39	Prolactinoma	〇	S and T
9	F	19	55	Pituitary adenoma	〇	T
10	M	25	49	Transection of the pituitary stalk	X	G, S, and T
11	F	33	36	Craniopharyngioma	〇	S, T, and A (+gamma knife)
12	M	33	59	Sheehan syndrome	X	S and T
13	M	34	42	Craniopharyngioma	〇	S, T, and A
14	M	58	75	Prolactinoma (+radiation)	〇	S and T
15	M	2	23	Idiopathic hypopituitarism	〇	G, S, and T

A, antidiuretic hormone; C, glucocorticoid; G, growth hormone; T, thyroid hormone.

**Table 2:** Comparison between surgical cases and nonsurgical cases.

	Surgical	Nonsurgical	*p* value
No.	8	7	
Female (%)	37.5%	4.3%	ns
Diagnosis age of hypopituitarism	16 ± 18.9	10.6 ± 10	ns
Diagnosis age of liver biopsy	40.3 ± 19.0	38.0 ± 16.1	ns
BMI	30.2 ± 4.1	29.2 ± 14.2	0.072
Obesity (BMI > 25)	87.5%	50%	0.067
Obesity (BMI > 30)	50%	14.3%	ns
Diabetes mellitus	75%	28.6%	0.072
Hypertension	50%	28.6%	ns
Dyslipidemia	75%	85.7%	ns

Data are mean ± SD or percentages. ns, not significant. BMI, body mass index.

**Table 3:** Comparison of blood tests between surgical cases and nonsurgical cases.

	Surgical	Nonsurgical	*p* value
Total bilirubin (mg/d)	0.7 ± 0.4	0.7 ± 0.3	ns
Albumin (g/dl)	3.9 ± 0.7	4.5 ± 0.3	0.054
AST (IU/L)	82 ± 39	94 ± 132	ns
ALT (IU/L)	72 ± 29	158 ± 258	ns
gGTP (IU/ml)	185 ± 64	156 ± 114	ns
Total cholesterol (mg/dl)	190 ± 27	241 ± 38	0.029
Triglyceride (mg/dl)	156 ± 64	227 ± 156	ns
Platelet (×104/ml)	13.6 ± 6.5	26.2 ± 6.0	0.002
Prothrombin time (%)	78.5 ± 13.3	89.3 ± 14.5	ns
HbA1C (%)	7.1 ± 2.5	5.4 ± 0.6	ns

Data are mean ± SD. ns, not significant.

**Table 4:** Comparison of liver pathological findings between surgical cases and nonsurgical cases.

	Surgical	Nonsurgical	*p* value
Fibrosis (F0–F4)	3.75 ± 0.38	1.64 ± 1.07	0.01
Inflammation (A0–A3)	1.17 ± 0.49	2.06 ± 0.73	ns
Steatosis (S0–S3)	2.31 ± 0.88	1.86 ± 0.90	ns

Data are mean ± SD. ns, not significant.

## Data Availability

No data were used to support this study.
